# Supplementary figures and images for: In vitro HIV DNA integration in STAT3 drives T cell persistence—A model of HIV-associated T cell lymphoma
Source: PLoS Pathog. 2025 Jul 8;21(7):e1013087. doi: 10.1371/journal.ppat.1013087 (PMC12251285; doi:10.1371/journal.ppat.1013087)

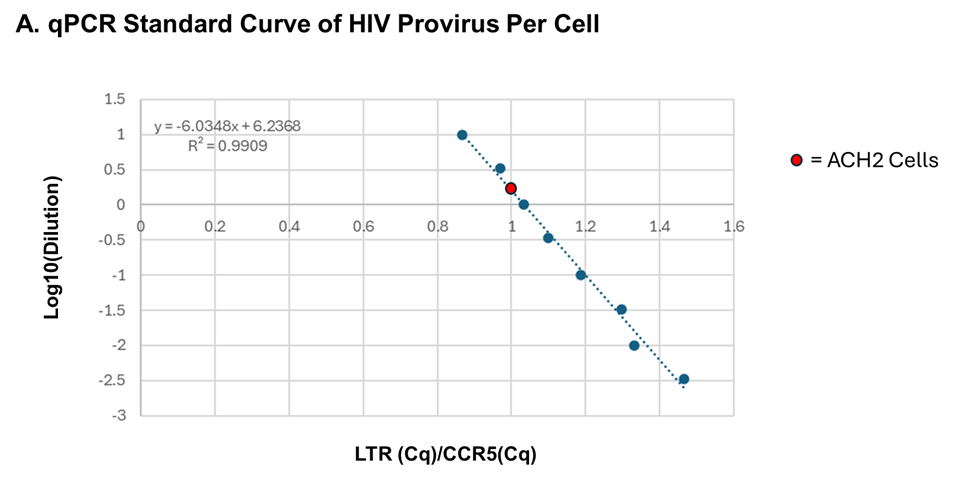

Supplement: S1 Fig — Masses of HIV-vector plasmid and cellular genome were calculated to determine an estimate of the number of proviruses per cell. The standard curve used mixtures of plasmid and Raji cell DNA starting at 10 proviruses per cell and diluted to 0.0033 proviruses per cell. X-axis is the Cq value of the LTR primer divided by the host gene CCR5 primer. Y-axis is the Log10 of the dilution. ACH2 cells are indicated by the red dot. (TIF) [file ppat.1013087.s001.tif]

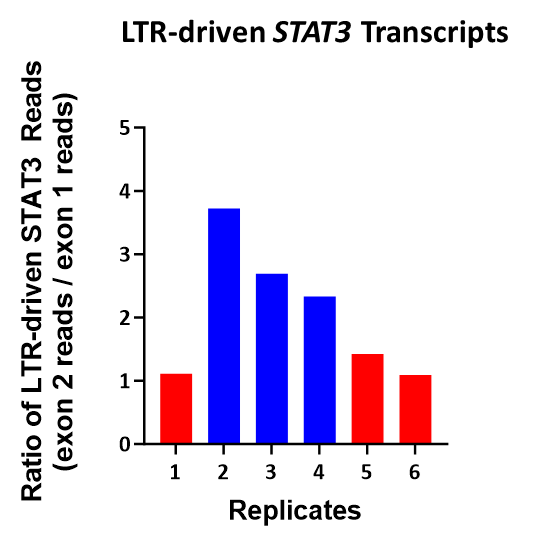

Supplement: S2 Fig — Bulk RNA-seq of unsorted donor 4 replicates was performed at day 126 post-transduction. Reps 2,3, and 4 were designated as GFP-High (Blue) replicates and Reps 1,5, and 6 as GFP-Low (Red). The ratio of coverage of STAT3 exon 2, downstream of the integration site, to that of upstream exon 1 in each replicate (see Fig 3B) allowed us to identify proviral driven transcripts in STAT3. (TIF) [file ppat.1013087.s002.tif]

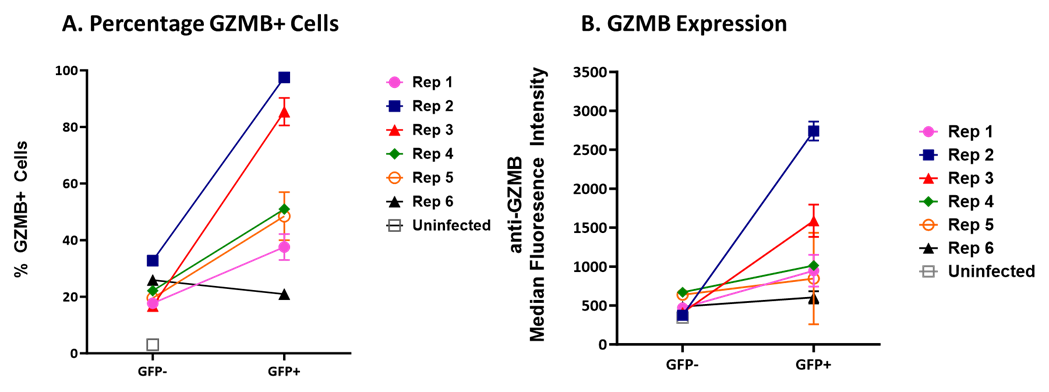

Supplement: S3 Fig — Flow cytometry of Donor 4 day 126 replicates was used to analyze GZMB expression. Cells were gated on viability using Zombie Violet. Cells were then gated on GFP expression and Frequency of GZMB positive cells was compared between GFP+ and GFP- cells of each replicate. (TIF) [file ppat.1013087.s003.tif]

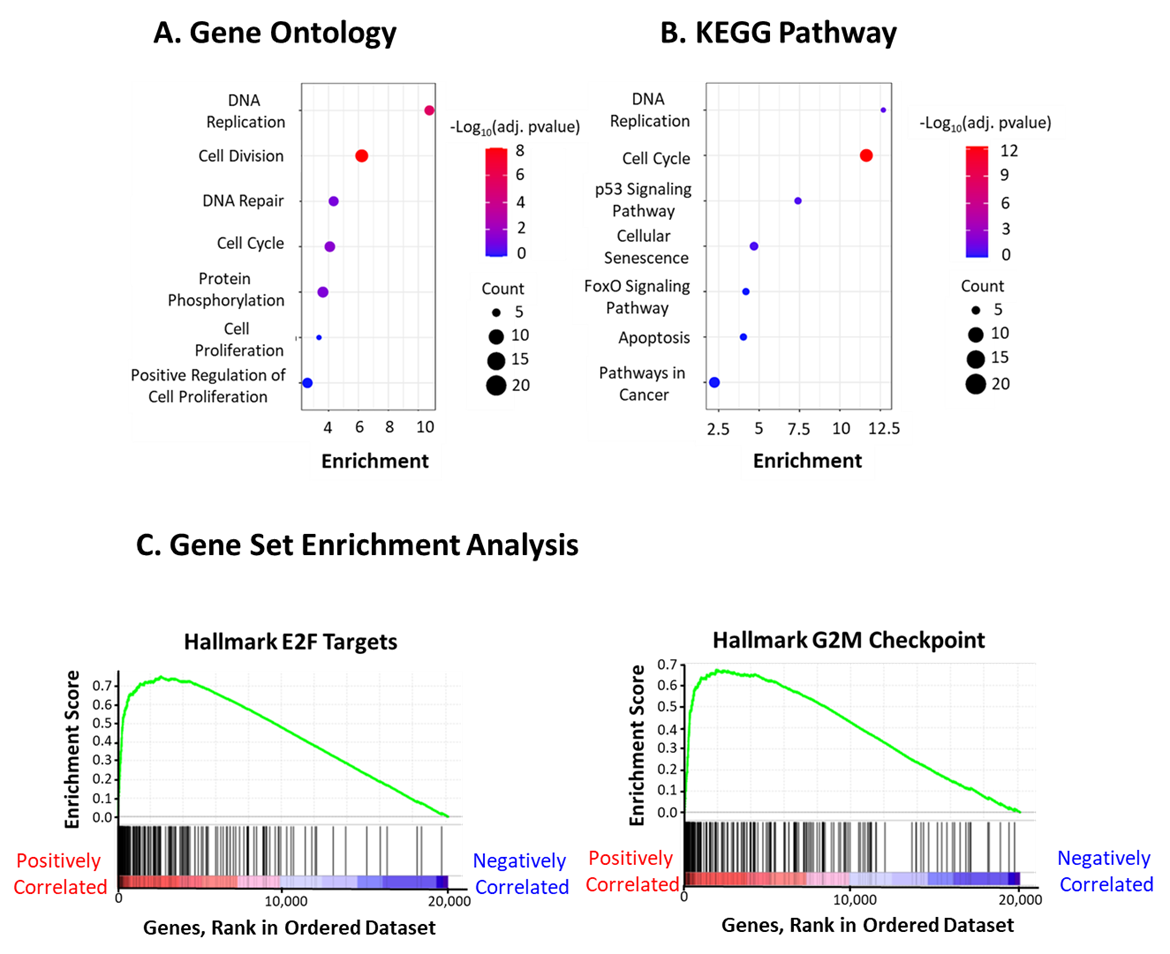

Supplement: S4 Fig — GFP-High replicates were sorted on GFP expression. Pooled RNA-seq data from the GFP-High replicates 2, 3, and 4 were used for subsequent analyses. A) Gene ontology analysis was performed using DAVID [100 ,101] on differentially expressed genes comparing GFP+ cells to GFP- cells. B) DAVID was used to compare KEGG Pathways. C) Normalized cell counts were used for GSEA to identify gene set enrichments between GFP+ and GFP- cells. FDR q-Value <0.001 for both gene sets. (TIF) [file ppat.1013087.s004.tif]

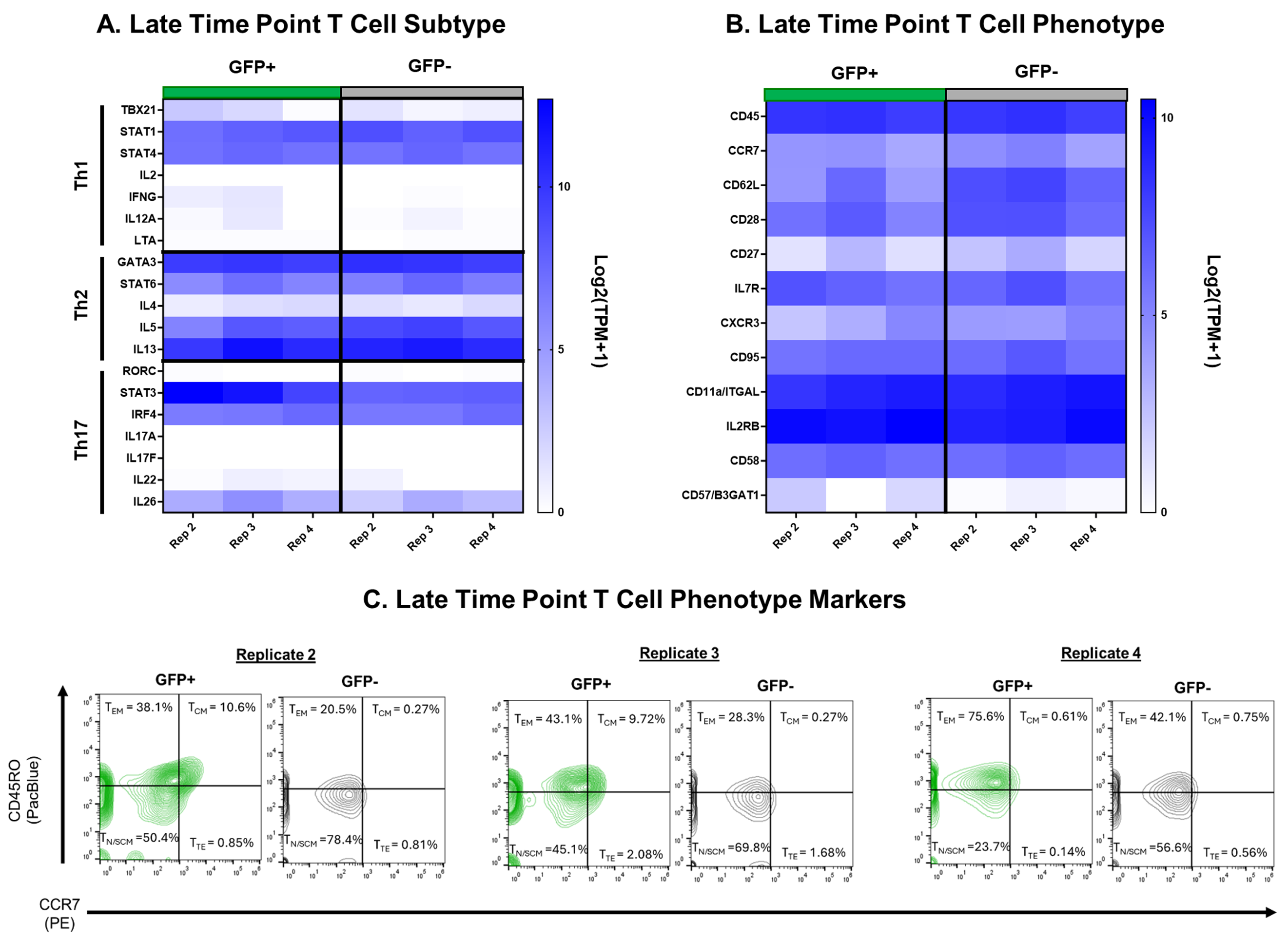

Supplement: S5 Fig — A-B) RNAseq data from sorted GFP-High replicates were used to determine expression of genes associated with different T cell subtypes and naïve, memory or effector T cell phenotypes. C) Flow cytometry was performed on late time point GFP-High replicate cells. Cells were gated on GFP expression and stained for CD45RO (PacBlue) and CCR7 (PE) to differentiate T cells from naïve and different memory phenotypes. (TIF) [file ppat.1013087.s005.tif]

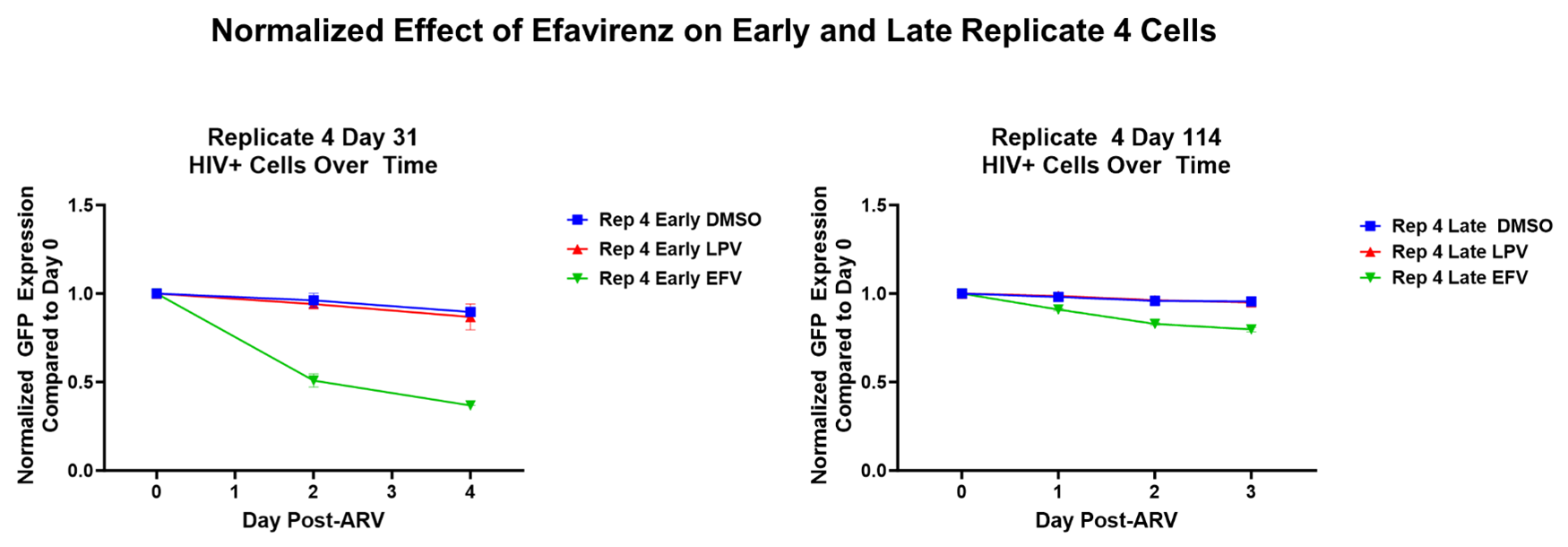

Supplement: S6 Fig — Replicate 4 cells at day 31 and 114 time points were assessed for GFP expression over short time periods with different treatment conditions of DMSO, 5μM LPV, or 5μM EFV. Early and late time point replicate 4 cells were assessed for GFP expression over the course of 96hrs and 72 hrs, respectively, with different culture conditions. GFP was normalized to pre-treatment GFP percentage and used to compare the effects of the different conditions. GFP was used as a reporter for HIV+ cells. (TIF) [file ppat.1013087.s006.tif]

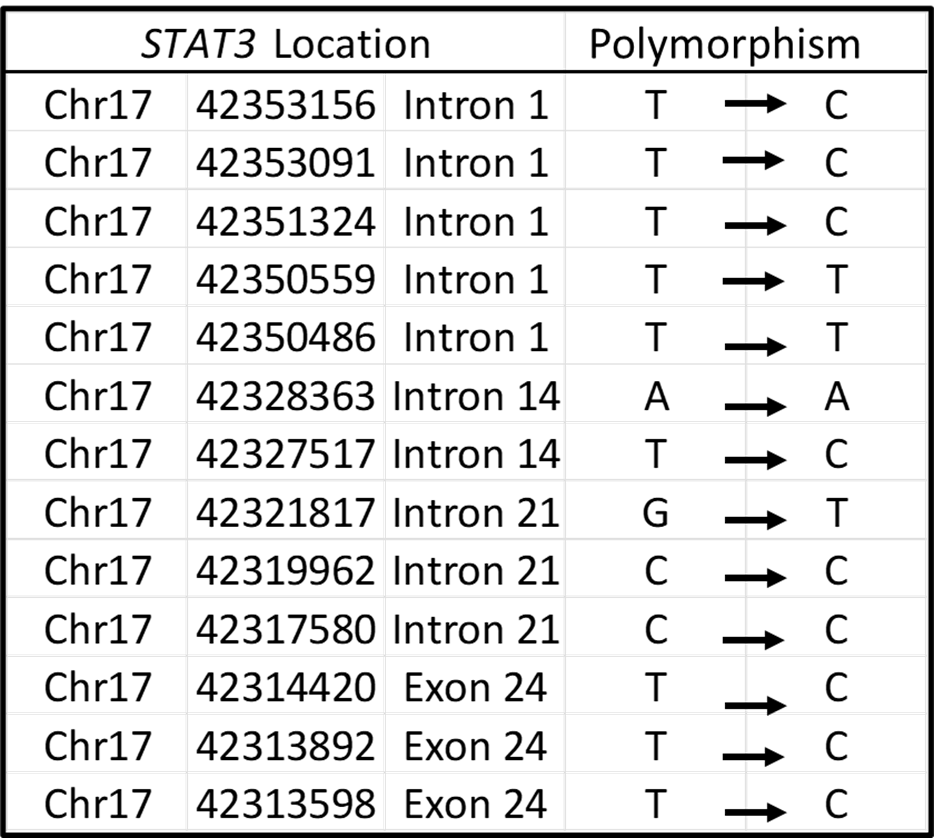

Supplement: S1 Table — A “call variants” analysis was used to compare STAT3 transcripts from Donor 4 replicates to the hg38 consensus STAT3 sequence. Individual nucleotide mutations are identified in the table. All mutations occur in untranslated regions of STAT3. (TIF) [file ppat.1013087.s007.tif]
