# Supplementary material for: In vitro HIV DNA integration in STAT3 drives T cell persistence—A model of HIV-associated T cell lymphoma
Source: PLoS Pathog. 2025 Jul 8;21(7):e1013087. doi: 10.1371/journal.ppat.1013087 (PMC12251285; doi:10.1371/journal.ppat.1013087)
Supplement: Attachment — Submitted filename: PLOS Pathogens Rebuttal Final.docx [file ppat.1013087.s010.docx]

PLOS Pathogens Point-by-point

First and foremost, we would like to thank all the reviewers for taking the time to review our manuscript. We believe that the feedback provided has led to edits and follow-up experiments that have made this a more compelling and impactful paper.

**Reviewer 1:**

Thank you for your encouraging feedback regarding our manuscript and scientific findings. We appreciate the comments and feel that they have given more clarity to the work we are presenting in this manuscript.

Major Issues

**Given that these are primary cells from uninfected donors (in vitro), not patient cells (ex vivo), please change “ex vivo” to “in vitro”.**

Thank you for recommending the change in how we address our model. We understand how using ex vivo would be confusing, as these cells are not from HIV+ individuals. We have changed all the “ex vivo” to “in vitro” including in the title of the manuscript.

Minor Issues

**Can the authors add leading edge genes to Figure 3E GSEA – is STAT3 one of the leading edge genes?**

Thank you for this suggestion, as it provides more clarity as to which enriched genes are associated with canonical STAT3 signaling. The list of leading-edge genes and their gene rank can be found in an updated Figure 3, specifically Figure 3F.

**“Additionally, insertional mutagenesis of STAT3 driven by the HIV proviral LTR was confirmed through detection of spliced HIV-STAT3 chimeric transcripts initiated in the 5’ LTR.” For HIV-driven splicing into host RNA, the authors may want to cite Cesana Nature Comm 2017, Liu Science Translational Medicine 2020, and Christian ML J Immunology 2022.**

Thank you for this recommendation and for catching this oversight. Please find that the recommended citations have been included on Line 160.

**Reviewer 2:**

We appreciate the encouraging words about the importance of this manuscript. Additionally, thank you for the inquisitive insights into how to improve the findings of this paper. Your experimental suggestions yielded an exciting finding, which we believe strengthens the significance of this manuscript.

**There is a discrepancy in the location of the integrant in the opposite orientation of GSDMD in replicate 4. In the main text the integrant is in exon 2, but in the table 1 it is reported as being in intron 2. I checked the location chr8:144641746 and it indeed looks like being in intron 2, according to hg19. Please verify and correct accordingly. This is relevant in my next point.**

We greatly appreciate you for catching this oversight. We missed this typo in our paragraph and have corrected it. Please find this correction on Line 458.

**I find the impact of the integrant in GSDMD particularly interesting, in light of the recent discoveries surrounding the inflammasome protein CARD8, which can sense HIV protease, leading to inflammasome activation and cell death by pyroptosis. GSDMD is the main effector molecule downstream of the CARD8 inflammasome in CD4+ T cells (in which IL-1beta and IL-18 are not activated, in the absence of other PAMPs/DAMPs). Given that CARD8 might sense HIV protease activity upon latency reversal and trigger cell death, if the integrant in GSDMD negatively affects Gasdermin D function, it would provide a survival benefit. In the Authors’ culture, there are no NNRTI that would enhance this effect (EFV and RPV cause premature protease activation in the cytoplasm, rather than after viral budding), but some spontaneous protease activity can occur. The Authors should discuss this process and the possibility that affecting Gasdermin D, GFP+ cells might be spared by some negative selection due to HIV expression. If they still have frozen cells from this replicate, the Authors could repeat the culture with and without 5uM of EFV and see whether the integrant in GSDMD has an even stronger survival advantage.**

Thank you for this insightful and exciting experimental suggestion. We cultured pre-clonal and clonal replicate 4 T cells in 5uM of EFV and found that the clonal time point (Day 114) showed resistance to EFV-mediated cell death compared to the pre-clonal or early time point from replicate 4. This is a very exciting finding as it shows evidence of how proviral integrations can influence survival. However, the focus of our manuscript is on integrations in STAT3 and how they model rare HIV+ lymphomas, and we feel that further exploration into how proviral integrations within *GSDMD* affect cell survival would be for a separate manuscript. We placed our findings within Supplemental Figure 6 and discussed the findings on lines 463-479 and 574-582. Thank you again for this novel idea which resulted in a very exciting finding that strengthens the impact of this study and sets up future avenues to utilize this in vitro model to study proviral integrations.

**The HIV construct used for their model is envelope and nef defective. I suggest to expand the discussion on the impact of the lack of these two genes, since Env can be cytopathic, and Nef can dampen T cell activation and reduce activation-induce cell death. The latter process has been studied recently in CAR-T cells (Perica et al., 2025), in which Nef expression extends their survival in vivo https://www.nature.com/articles/s41586-025-08657-0.**

We appreciate this insightful point about discussing how the use of our HIV construct might influence T cell survival or growth. We feel that this suggestion provides more clarity to our model and allows us to discuss more limitations of this in vitro model. Please find our discussion of our HIV construct and limitations of the model on lines 615-625.

**The sentence in lines 157-159 is convoluted. Please simplify the description of the experimental setup.**

Thank you for identifying this area that needs clarification. Please see our updated description of the experimental set up now located on lines 153-156.

**I understand what you mean in line 189: “CCR5 is a single-copy cellular host gene”. But please clarify that it’s still a diploid gene. SRY on the Y chromosome is an example of a true single-copy gene.**

Thank you again for identifying another area that needs more clarification. Please find the correction stating that CCR5 has two copies in a diploid genome on Lines 192-193. This suggestion strengthens the explanation of our experimental design. SRY would have been a good gene to use as a single-copy standard, but, unfortunately, donor 4 was a female.

**Could the Authors speculate, in the discussion, which experiments should be attempted in the future to better assess the impact on T cell survival/proliferation of integrants in genes with unclear role in T cells? Such as CCDC77 and DCUN1D4?**

We greatly appreciate this comment, as it adds value to our discussion section. As CCDC77 and DCUN1D4 are poorly characterized genes, discussing future experiments to identify their role in survival is important. We now propose overexpressing these sequences in primary T cell cultures in the presence or absence of a provirus in *STAT3* to determine how they might influence T cell growth or survival. This addition will give those reading our study ideas to better understand the role these genes play in T cell biology and cell survival or proliferation. Please find our discussion suggestions on lines 570-573.

Reviewer 3:

Thank you so much for your insightful suggestions on how to improve our manuscript. We appreciate your recognition of the significance of this study as well as your perspective on the areas in which it can be improved. Your suggestions were taken with careful consideration and we feel that they have strengthened the impact of our study.

Major Issues

**In my experience the ACH-2 cell line has a higher level of proviral DNA per genome (~4 copies of HIV/ diploid genome) than reported in this study. Notably, a study from Symons et al demonstrated that ACH-2 cell line has approximately ~100 unique integration events [2]. In fact, this study demonstrated that the number of integration sites increases over time in culture due to ongoing replication.
I recommend the authors carefully calibrate the level of integration in this cell line. For example, they could perform limiting dilution PCR on purified DNA from a precisely quantified number of cells to determine the HIV copy number. This is relevant to the conclusions of Figure 6 A. In this Figure, the authors conclude that replicates 2,3, and 4 have between 1 and 1.5 integrations per cell. This conclusion assumes their ACH-2 control cell line has 1 integration per cell on average. However, if the ACH-2 cell line has 2 or more integrations per cell then the average number of integrations per cell in their ex vivo culture is likely higher than 3 per cell. In this scenario, it seems likely that multiple integrations may play a role in whether a clone expands. While the potential role of additional integration sites is acknowledged in the paper, carefully calculating the average number of integrations per cell informs the reader of the likelihood of this confounder.**

We greatly appreciate this acknowledgement of a weakness in our manuscript. This insight has greatly improved the take-aways from our manuscript and provides even more insight in how proviral integrations may influence T cell growth, survival and oncogenesis.

Using this feedback we confirmed the number of proviruses per ACH2 cell using a standard curve of our HIV vector diluted in cellular DNA. Based on the mass of the vector and cellular genome we diluted to get a curve of 10 proviruses per cell to 0.001 proviruses per cell. Using this curve, we have determined that the ACH2 cell DNA we used has about 2 proviruses per cell. Please find our standard curve in Supplemental Figure 1. Based on these findings we have updated the calculations in our manuscript, which now show 2 or more than proviruses d within each of the gfp+ clonal T cell populations.

Please find an updated discussion on lines 187-195, 436-437, and 548-552. Figures 1C and 6A have been updated accordingly.

**In their model, the frequency of intergenics increases over time from 41% to 57% (assuming that the sites identified by “.”are intergenics, 2911 out of 5807). Prior studies indicated that the majority of integration sites are in genes when assayed 48 hours after infection of a cell line [3] or when detected in vivo [4]. Can the authors discuss their findings in the context of this literature?**

Thank you for acknowledging this aspect of our manuscript and allowing us the opportunity to clarify our findings. This comment therefore strengthens our findings and validates previous findings in the literature.

We agree with your comment that previous findings show the majority (ca 80%) of integrations that occur immediately after transduction occur in genic regions of the genome. Our earliest (day 3) DNA was unavailable for this analysis, so therefore we used the next time point Day 17, where some decline in genic integration had already occurred. We still see this decline in integrations within genes, which agree with in vivo findings by Coffin et al, PLOS Path, 2021. We hope that clarification of this point further strengthens our paper and understanding of the role of integrations within genes is largely negative but beneficial if it leads to insertional mutagenesis of a functional gene product that contributes to survival.

Discussion and updates can be found on lines 231-235, and 522-527

**Given the role of STAT3 in T cell development, it would be interesting to compare the T cell phenotype of the T cell clones with HIV integrated in STAT3. If this is not feasible, a strong surrogate might be to compare the T cell phenotype of the GFP+ and GFP- cells at late time points (~day 150).**

Thank you so much for this excellent suggestion. We were also very interested in the question of whether STAT3 overexpression influences T cell subtype and/or phenotype due to the role of STAT3 in T cell development. We initially left out our data because we felt that it is difficult to come to any conclusions due to the in vitro environment of our cell growth. Given the interest of the reviewer we have included our data in supplemental figure 5. We found that our T cells did not change into a Th17 subtype, associated with STAT3 expression in vivo, by utilizing our RNAseq data. Additionally, our findings support previous findings that naïve T cells that do not receive a secondary signal present as a Th2-like phenotype.

Additionally, our RNAseq data did not reveal any distinct differences in memory phenotype but our flow data confirmed an increase in memory T cells formation. However, not all the replicate 2 GFP/HIV+ cells showed this change. However, the Gfp-positive populations in all of the GFP-High replicates did skew towards a memory phenotype with replicates 2 and 3 that contained more cells with proviral integrations in STAT3, had more central memory -like T cells compared to replicate 4 and the GFP-negative populations. This increase in memory phenotype could be one of the contributors of HIV+ persistence.

Our data can be found in supplemental 5 and discussed on lines 384-404 and 615-621.

We hope that these data and discussion points are enough to answer your question. Thank you again for the suggestion as this strengthens the findings of our paper and we expect it to improve the impact of our research. The findings that STAT3 overexpression by insertional mutagenesis improves T cell memory phenotype differentiation is an impactful finding. Additionally, that selection for this phenotype in HIV+ cells helps further the field of defining the events that contribute to maintenance of HIV reservoir.

We have added a sentence to the Acknowledgments thanking all 3 reviewers for their contributions to strengthening our manuscript.
